# Barriers and facilitators to implementation research on pharmacist-led medication reviews in memory clinics: A qualitative study using the TDF-COM-B

**DOI:** 10.1371/journal.pone.0341014

**Published:** 2026-01-20

**Authors:** Rishabh Sharma, Yusra Aslam, Sadaf Faisal, Carrie McAiney, Feng Chang, Cheryl A. Sadowski, Linda Lee, Manonita Roy, Bincy Baby, Muhammad Mamdani, Tejal Patel

**Affiliations:** 1 School of Pharmacy, University of Waterloo, 10 Victoria St S A, Kitchener, Ontario, Canada; 2 School of Public Health Sciences, University of Waterloo, Waterloo, Ontario, Canada; 3 Schlegel-UW Research Institute for Aging, 250 Laurelwood Dr, Waterloo, Ontario, Canada; 4 Faculty of Pharmacy & Pharmaceutical Sciences, College of Health Sciences, University of Alberta, Edmonton, Alberta, Canada; 5 CFFM MINT Memory Clinic, 16 Andrew Street, Kitchener, Ontario, Canada; 6 Department of Family Medicine, McMaster University, 100 Main St W Floor, Hamilton, Ontario, Canada; 7 Division of Geriatric Medicine, Department of Internal Medicine, University of Ottawa, Ottawa, Ontario, Canada; 8 Institute of Health Policy, Management, and Evaluation, University of Toronto, Toronto, Ontario, Canada; 9 Li Ka Shing Knowledge Institute, St Michael’s Hospital, Toronto, Ontario, Canada; 10 Leslie Dan Faculty of Pharmacy, University of Toronto, Toronto, Ontario, Canada; 11 University of Toronto Temerty Centre for Artificial Intelligence Research and Education in Medicine (T-CAIREM), Toronto, Ontario, Canada; No institution, UNITED KINGDOM OF GREAT BRITAIN AND NORTHERN IRELAND

## Abstract

**Background:**

Improving medication use is important in patients with cognitive impairment and dementia. In memory clinics within primary-care settings, pharmacists perform structured medication reviews targeting medication-related problems in this population. The impact of pharmacist-led medication reviews in such clinics has not been examined previously. However, implementing research in clinical environments is fraught with challenges. Thus, this research focuses on identifying barriers and facilitators that would impact the implementation of research projects examining medication reviews in primary-care based memory clinics.

**Methods:**

We conducted a qualitative study using focus group discussions with pharmacists, physicians, care partners, and other healthcare professionals. Semi-structured guides informed by the Theoretical Domains Framework (TDF) were used to facilitate discussions. Using the TDF–COM-B (Capabilities-Opportunities-Motivation-Behaviour) approach, we identified and analyzed barriers and facilitators to implementing research on pharmacist-led medication reviews and generated potential intervention strategies.

**Results:**

Eighteen stakeholders across three focus groups identified several key barriers, including communication gaps, unclear interdisciplinary roles, participant burden, and limited resources across multiple TDF domains. Facilitators included the perceived impact of medication reviews (beliefs about consequences), patient involvement in research (decision processes), study procedural knowledge (knowledge), pharmacist recognition (social/professional role and identity), and standardized medication reviews (skills). Implementation strategies included providing standardized training, simplifying study procedures with digital tools and templates, involving study coordinators, and engaging patients and care partners in decision-making. Barriers such as communication gaps, unclear interdisciplinary roles, participant burden, and resource constraints were addressed through workflow redesign, financial incentives, and fostering collaboration and institutional support.

**Conclusion:**

This study identified key barriers and facilitators to implementing research on pharmacist-led medication reviews in memory clinics, highlighting several areas with direct policy relevance. These findings point to important policy needs, including clearer interdisciplinary roles, improved communication, adequate resource support, and standardized training to enhance the feasibility and sustainability of pharmacist-led medication review interventions, ultimately supporting successful research implementation and improving medication use in older adults with cognitive impairment and dementia.

## 1. Introduction

Dementia impacted almost 50 million individuals globally in 2017, and that figure is expected to rise to 152 million by the year 2050 [1]. Older adults with dementia often have a high prevalence of comorbidities and are more likely to experience polypharmacy [2]. Polypharmacy is also associated with the use of potentially inappropriate medication (PIMs), a risk of adverse events, and drug interactions [3]. Polypharmacy also increases the complexity of medication regimens, resulting in declining medication adherence [4]. Drug-related problems (DRPs) are a significant concern in the care of community-dwelling older adults living with cognitive impairment and dementia [5]. Furthermore, DRPs in older persons with cognitive impairment or dementia (PWCID) can lead to an increased risk of hospitalization, decreased quality of life, healthcare costs, risk of falls, medication non-adherence, and mortality [6,7]. Research indicates 66–93% of people with dementia experience at least one DRP [5,8].

Due to these DRPs, optimizing medications in PWCID is of great significance [9]. A medication review is a critical assessment of an individual’s medications that aims to improve the quality, safety, and appropriate use of medications [10]. Pharmacists play a crucial role in conducting medication reviews by addressing problematic DRPs, reducing polypharmacy, and mitigating PIMs use among older adults, including those with cognitive impairment or dementia [11]. Their expertise in medication management and ability to collaborate with other healthcare professionals make them valuable members of interdisciplinary primary-care-based memory clinic teams.

Overall, pharmacist-led community-based medication reviews for older adults improve outcomes by decreasing DRPs and improving medication adherence [12,13]. Among PWCID, pharmacists’ interventions have been shown to decrease the number of inappropriate medications after a medication review. For example, Pearson et al. reported a decrease in the mean number of PIMs in PWCID from 1.5 PIMs per patient at baseline to 0.9 PIMs per patient at 180-day follow-up [14]. In Europe, comprehensive medication reviews conducted by pharmacists significantly improved the quality of medication use [15]. In this study, PIMs use in the intervention arm decreased from 20.3% to 14.2% (p = 0.002); specifically, use of anticholinergic medications decreased from 7.1% to 3.3% (p = 0.005), and the use of non-steroidal anti-inflammatory drugs (NSAIDs) decreased from 3.3% to 0.9% (p = 0.025) [15].

However, previous research on medication review in PWCID indicates that medication reviews vary in scope and depth, are frequently not structured nor comprehensive, and do not reflect interdisciplinary team environments [16,17]. In Canada, pharmacists who practice in primary-care-based interdisciplinary memory clinics, known as the Multispecialty Interprofessional Team (MINT) Memory Clinics, are trained to conduct structured medication reviews, focusing on DRPs frequently encountered in PWCID [18]. As of 2024, over 100 such clinics have been established in parts of Ontario, Saskatchewan, Alberta, British Columbia, and New Brunswick to assess, diagnose, and provide care to PWCID [18]. This model focuses on building capacity within primary care to identify, assess, and manage cognitive disorders through interdisciplinary collaboration [19–22].

The impact of pharmacist-led medication reviews in these clinics has not been examined previously. The implementation of a large-scale clinical trial may be challenging in the context of varying organizational and operational barriers. Identifying barriers and facilitators before attempting a large study is crucial for designing targeted interventions to address these challenges a priori, optimizing resource allocation, setting realistic expectations, addressing ethical considerations, engaging stakeholders, mitigating risks, and enhancing intervention implementation. Without this exploration, the risk of encountering unforeseen challenges during the study increases significantly. This study was a part of a larger project which had three aims: (1) to examine stakeholder perspectives on medication use, burden and management of persons with dementia; (2) collaboratively generate and prioritize the top 5 research questions about medication use and reviews and (3) identify barriers and facilitators that may impact the research related to medication reviews in primary-care based memory clinics. The current study was designed to identify barriers and facilitators that may impact the implementation and conduct of research evaluating pharmacist-led medication reviews in primary-care-based memory clinics.

## 2. Methods

### 2.1. Study design

We conducted a qualitative study using focus groups. The focus group discussions took place in a combination of in-person sessions held at the School of Pharmacy, University of Waterloo, and virtual sessions conducted via secure videoconferencing. Participants did not have a pre-existing relationship with the study setting. They were recruited from different memory clinics and organizations across the region, resulting in mixed groups rather than clinic-specific groups.

### 2.2. Theoretical underpinnings

A combination of the Theoretical Domains Framework (TDF) and the COM-B model (Capability, Opportunity, Motivation–Behavior) was used in this study. The TDF is a widely used framework in behavioral science and implementation research [23]. It provides a comprehensive approach to understanding and analyzing the factors influencing behavior change and adopting new practices. The TDF helps researchers and practitioners identify barriers and facilitators to behavior change, which is particularly valuable when designing interventions or strategies to improve healthcare practices, including medication management [23]. TDF is divided into 14 domains, developed to identify and understand the factors influencing behavior change and implementing evidence-based practices in various fields, including healthcare, public health, and social sciences [23,24]. Each domain further consists of a broad category of theoretical constructs. Theoretical constructs within the TDF represent the abstract concepts or variables derived from established psychological or behavioral theories and provide a holistic understanding of the factors influencing behavior change, helping researchers and practitioners design effective interventions tailored to specific contexts. Furthermore, the 14 TDF domains are mapped to COM-B (Capabilities-Opportunities-Motivation-Behaviour system within Behavior Change Wheel (BCW)), which informs the design of enhanced implementation strategies [25]. The BCW is an implementation framework centered around Capability (C) (i.e., physical or intellectual ability to perform the task), Opportunity (O) (i.e., factors in the physical and social environment that make a behavior possible), and Motivation (M) (i.e., reflective processes or automatic impulses or feelings that drive actions). When developing the interview guide for the focus group including patients/care partners, we did not include TDF domains relating to professional role, integration, and implementation. These domains were deemed less applicable to the focus group participants, as they are more relevant to clinical practice and organizational processes typically addressed by healthcare professionals such as pharmacists, physicians, and other clinicians. Therefore, these domains were only explored within the Clinician (PH and HCP) focus groups.

### 2.3. Recruitment and sampling

We used a purposive sampling strategy, supplemented by convenience sampling, to ensure representation from key stakeholder groups involved in memory clinic care and medication management. We invited persons with CI and dementia and informal care partners, family physicians and geriatricians, pharmacists, community support workers, nurses, social workers, occupational therapists, and researchers through the networks of MINT Memory clinics and professional relationships between October 19th, 2022, and January 19^th^, 2023. These groups were purposefully selected because each contributes unique perspectives on medication use, workflow processes, and implementation challenges in memory clinic settings. The study was publicized via social media, including the University of Waterloo School of Pharmacy’s website and X (formerly known as Twitter) account, as well as through the researchers’ networks and by reaching out to previous study participants who had indicated an interest in participating in future research initiatives. Participants could participate in the study if they were: (1) individuals diagnosed with dementia or cognitive impairment; (2) care partners directly involved in the medication management of PWCID on a daily basis; or (3) healthcare professionals, including physician, pharmacist, nurse, social worker, occupational therapist, community social worker in Canada; (4) healthcare administrators. Physicians were required to hold an active license and have direct patient contact in a memory clinic or related geriatric setting, with no minimum years of experience required. Pharmacists were not required to be practicing in a memory clinic but needed prior experience conducting medication reviews for older adults. Care partners were unpaid family members or spouses who were directly involved in the daily medication management of the PWCID. To provide background and context, stakeholder participants were invited to attend two virtual sessions before participating in the interviews. During these virtual sessions, trainees, and national and international researchers in the field of geriatrics presented on: (1) the MINT Memory Clinic model; (2) the pharmacist role within this model; (3) DRPs frequently encountered in persons with cognitive impairment; (4) the definition, purpose and components of a medication review; (5) instruments used to conduct medication reviews in PWCID; (6) core outcomes for studies examining the effectiveness of medication reviews and interventions in PWCID and; (7) sex and gender based analysis. Following these virtual sessions, participants were invited to attend a day-long session either in person or virtually. All participants received the same introductory information regardless of their assigned focus group. Additionally, the same participants were involved in a storytelling session, followed by a session utilizing a nominal group technique to build consensus and prioritize research questions exploring medication use in Memory Clinics in the first half of the day. In the second half of the day, participants were divided into small groups of 5–6 to participate in three focus groups, where we identified barriers and facilitators for implementing research in Memory Clinics. Although data saturation was not reached, the final sample size is appropriate for qualitative focus group research, where the aim is to capture diverse perspectives rather than achieve numerical representativeness. The sample included a wide range of stakeholder groups, providing rich insights into medication review implementation. The targeted number of participants was not reached because several individuals declined due to scheduling and workload constraints, and some did not respond to invitations.

### 2.4. Data collection

The three focus groups were facilitated on January 20, 2023, by three clinical pharmacist researchers (TP, FC, and SF) serving as the primary facilitators, with a co-facilitator assigned to each group (CM, LL and CS). Each discussion lasted approximately one hour. Two facilitators (FC and TP) have over 20 years of experience in both clinical pharmacy practice (with Bachelor of Science in Pharmacy (BScPharm) and Doctor of Pharmacy (PharmD) degrees) and research while one facilitator (SF) was a post-doctoral researcher who has more than 15 years experience as a pharmacist (with a Bachelor’s of Pharmacy (BPharm)) and Doctor of Philosophy (PhD) degrees and was trained in qualitative research methods. Each facilitator was assisted by research assistants (BB, YA, RS) who were undergraduate or graduate students. These research assistants were responsible for taking notes during the meeting, audio recording the conversations, and transcribing the interviews and discussions. They were not involved in the facilitation or content of the discussions. All interviewers followed a standardized semi-structured focus group interview guide based on the TDF to ensure consistency and methodological rigor. The questions in the semi-structured focus group guide were developed based on the 14 domains of the TDF [23]. While the semi-structured interview guides in all three focus groups were based on the TDF, the interview questions were tailored slightly to each group to reflect their specific roles and experiences in medication review implementation. This structure allowed for a deeper understanding of the unique and shared barriers and facilitators across stakeholder groups. Questions covering each domain explored stakeholder perceptions of barriers and facilitators on the local operationalization of the research (e.g., ethics board approval, recruitment, data collection) (see supplementary tables, S1 Table and S2 Table). We used the consolidated criteria for reporting qualitative research checklist to guide reporting of this study (see Appendix). No individuals other than the participants and research team were present during the focus groups. Each participant attended a single session, and no repeat interviews were conducted. Transcripts were not returned to participants due to time and resource constraints, and participant checking of findings was not performed as this was an exploratory, single-day study. Thus, items 15, 18, 23, 29 were marked as not applicable in the checklist. Prior to participating in the focus groups, all participants received written and verbal information about the research project and provided written informed consent for both their participation and the audio recording of the sessions.

### 2.5. Data analysis

All the focus group sessions were audio recorded and transcribed verbatim using the NVivo transcription services (QSR International, Melbourne, Australia). All the focus group transcripts were checked for accuracy and anonymized. Two study team members (RS, YA) reviewed the transcripts for each focus group. One of the researchers (RS) is a graduate student with experience as a researcher and non-practising pharmacist. The other researcher (YA) is an undergraduate student in health sciences. Data analyses were carried out in two stages. The first stage involved an inductive coding approach to identify the emerging themes related to facilitators and barriers to conducting research on medication reviews in PWCID. Each transcript was coded line by line independently by two researchers (RS, YA). The codes were subsequently compared to identify any disagreements and to finalize and define the codebook, ensuring consistency and clarity in the coding process. Disagreements were resolved through discussion with a third researcher (SF), a practicing pharmacist in Canada with more than 15 years of practice experience, as well as extensive experience in qualitative research. The next stage involved a mapping of identified codes to the TDF domains and theoretical constructs within each domain; all codes identified from the inductive coding were deductively mapped to the appropriate TDF domain(s) and theoretical constructs. During the mapping process, codes were mapped based on their relevance to the TDF domains and constructs for each focus group. No comparison was conducted between different focus groups in this study. Two researchers also performed the deductive coding, and any disagreements were resolved through discussion, with involvement of the third researcher as needed. The discussions also encompassed broader aspects, such as refining the coding framework and ensuring alignment with the research objectives. We refined themes by classifying them into TDF domains related to each COM-B category. We then identified BCW strategies for the TDF domains identified.

### 2.6. Ethics approval

The research was granted ethical clearance by the Clinical Research Ethics Board at the University of Waterloo, Canada (ORE # 44618).

## 3. Results

Eighteen stakeholders were recruited. We aimed to bring together 30 stakeholders, including researchers, clinicians, care partners, older adults with dementia, and healthcare system administrators. Invitations to participate in focus groups- held either in person or virtually- were sent to 20 pharmacists, 8 physicians, 5 nurses, 3 social workers, 2 occupational therapists (OTs), 6 healthcare administrators, and 4 persons with dementia and/or their care partners. In total, 29 stakeholders attended the session, including 13 members of the research team (4 undergraduate students, 2 graduate students, 1 post-doctoral researcher, 3 pharmacists’ researchers, 2 physician researchers, and 1 gerontology researcher), as well as 7 pharmacists, 3 social workers, 2 occupational therapists, 1 healthcare administrator, and 3 care partners. The final composition of the focus groups was as follows: Focus Group 1 (CP) included 4 participants comprising 3 care partners and one healthcare administrator; Focus Group 2 (PH) consisted of 9 participants, including 7 pharmacists and 2 physicians; and Focus Group 3 (HCP) included 5 healthcare professionals—3 social workers and 2 occupational therapists. Three separate focus groups were conducted to ensure that perspectives from diverse stakeholder roles were captured without influence from professional hierarchies or group dynamics that might inhibit open discussion. The first group included care partners and healthcare administrators (CP), who provided insights into caregiver responsibilities and system-level implementation challenges. The second group consisted of pharmacists and physicians (PH), offering clinical perspectives on workflow integration, professional roles, and prescribing practices. The third group included social workers and occupational therapists (HCP), who contributed viewpoints on patient support, communication, and interdisciplinary collaboration.

A detailed description of the demographic characteristics of included study participants is summarized in Table 1.

**Table 1:** Study participants demographic characteristics.

Focus group 1 (Carepartner and Health Care Administrator)				
**Number of participants**	**Sex n (%)**	**Age range**	**Helps with medication management n (%)**	**Level of Education**
CP = 3 HCA = 1	F = 4 (100%)	50- 74	Yes = 2 (50%)	Some college/university, no degree = 2 (50%) College/University degree = 2 (50%)
**Focus group 2 (Pharmacist/Clinician)**				
**Number of** **participants**	**Sex n (%)**	**Occupation**	**Work experience range**	
RPH = 7	F = 3 (43%) M = 4 (58%)	Pharmacist	2- 37 years	
Physician = 2	F = 2 (100%)	Geriatrician, Family Physician	10- 35 years	
**Focus group 3 (Healthcare professionals)**				
**Number of** **participants**	**Sex n (%)**	**Occupation**	**Work experience range**	
HCP = 5	F = 5(100%)	Registered Social Worker = 3 Occupational Therapist = 2	6- 31 years	

CP = Carepartner HCP = Healthcare Providers, HCA = Healthcare Administrators, RPH = Registered Pharmacist, F = Female, M = Male

Table 2 summarizes the barriers, facilitators, and implementation strategies for conducting pharmacist-led medication reviews in primary care-based memory clinics. It maps these codes to relevant theoretical domains within the TDF and connects them to the COM components of the BCW.

**Table 2:** Barriers, facilitators, and behavior change strategies for implementing pharmacist-led medication reviews in memory clinics: A COM-B and TDF-Informed Analysis.

COM-B	COM-B Subcategory	TDF Domain	Facilitators	Barriers	Intervention functions
Capability	Physical capability	Physical Skills	Specialized certification or training needed for Pharmacist- PH, HCP	Different challenges for different patient population- PH	**Education** 1. Develop educational workshops or webinars tailored to healthcare professionals’ roles, focusing on research and medication review methods 2. Informing participants about the study, emphasizing accurate data collection and the importance of the research 3. Providing multi-language handouts for patients and care partners to ensure study understanding 4. Use visual aids (e.g., flowcharts) to clarify complex study procedures) **Training** 1. Hands on training and real work scenarios that address both general and patient specific challenges (e.g., managing polypharmacy in older adults with dementia etc) 2. Provide hands-on training for healthcare professionals to practice study-related tasks, such as data entry and conducting medication reviews 3. Incorporate role-play exercise to address communication with patients and care partners, provide scenario-based training focused on managing difficult conversations (e.g., addressing medication non-adherence)) **Enablement** 1. User-friendly tools, such as template for data collection and flowcharts for medications reviews to reduce complexity and ensure consistency 2. Provide ongoing support, such as access to a study coordinator for clarifications 3. Implement feedback mechanisms (e.g., surveys or focus groups) to incorporate participant input into study processes) **Environmental Restructuring** 1. Set up dedicated training spaces with resources tailored for healthcare professionals 2. Provide study toolkits, including templates, checklists, and flowcharts for standardization) **Modelling** **1.** Showcase success stories of how professionals overcame expertise-related challenges to excel in their roles
			Skill on conducting research in pharmacy- HCP		
			Demonstrate competence in pharmacy work- PH		
			Fundamental requirement for HCP- HCP	Too much training limits its applicability- PH	
	Psychological capability	Knowledge	Standardized training for study procedures- PH, HCP		
			Education on accurately conducting the study procedures- HCP		
			Accurate data collection- HCP		
			Understanding of study procedures- HCP		
			Understanding the importance of research- How research will help memory clinic to improve- HCP		
			Provide information about the study to the patient and care partner- CP		
		Cognitive and interpersonal skills	Level of interaction with patient population- PH		
		Decision making	Patient and Care Partners part of the study and decision making- CP		
		Behavioral regulation	Questioning the expertise- PH		
Motivation	Reflective motivation	Social/professional role	Value of pharmacist role- HCP More pharmacy involvement or pharmacy capacity- PH Role of pharmacist in getting more resources- PH		**Education** 1. Develop training materials on aligning interdisciplinary goals and addressing patient-centered research areas, 2. Developed educational workshop showcasing evidence of pharmacist’s contributions to patient outcomes (e.g., fewer hospitalizations, improved medication adherence), cost savings, and system efficiencies 3. Seminars or workshops that provide data and case studies on successful pharmacist led initiatives 4. Offer training on advocacy and communication skills to help pharmacists articulate their value to other healthcare professionals and stakeholders 5. Guidelines and protocols to clarify roles and expectations for pharmacist participation, create educational material **Persuasion** 1. Use testimonials from patients, care partners, and healthcare professionals who have benefited from medication reviews to convey real-world impact 2. Highlight the pharmacist’s role in deprescribing and optimizing medication use through impact stories and videos 3. Highlight the cost-effectiveness of pharmacist-led interventions in interdisciplinary care through success metric 4. Use data visualizations to demonstrate measurable improvements in medication adherence, safety, or patient outcomes due to pharmacist participation 5. Emphasizing the pharmacist value in improving patient care and obtaining resources) **Modelling** 1. Showcase successful pharmacists who have excelled in expanding their role or securing resources in interdisciplinary teams 2. Role-play scenarios in training to practice managing complex cases and team interactions 3. Use peer-led demonstrations where professionals share their experiences and solutions for addressing differing perspectives **Enablement** 1. Develop checklists, templates, and decision-support tools for conducting medication reviews and documenting study findings 2. Create feedback loops where pharmacists receive constructive input on their contributions to the study 3. Streamline data collection processes with user-friendly tools or digital platforms to save time 4. Assign study coordinators or assistants to handle administrative tasks, reduce the workload for healthcare professionals 5. Incorporate flexible scheduling for study-related activities to minimize disruptions to regular workflows
		Professional identity	Recognition and Acknowledgment of pharmacist work in memory clinic- PH	Role of interdisciplinary staff in this study- HCP	
		Beliefs about capabilities	Positive effects of medication review- PH, CP Pharmacist participation will make the study easier- HCP		
		Beliefs about consequences: Beliefs	Medication review yields better outcomes/ quality of care- PH, HCP, CP Medication use experience- PH Evidence of effectiveness or benefits of medication review to the patient- PH, HCP, CP Tangible outcomes or benefits from the study will encourage patients to participate- PH, CP Positive effects of pharmacists in deprescribing and appropriate use of medications- PH, CP Proving the Value of interdisciplinary team facilitates funding- HCP Difference in care for people who visit FHT and Interdisciplinary team and memory clinics as compared to people in community setting- HCP Showing benefits of the medication review research will facilitate advancement and expansion of memory clinics- HCP		
		Beliefs about consequences: Outcome expectancies	Value of effective medication review (Prevent hospitalization; prevent premature placements, increase quality of life)- PH, HCP		
		Beliefs about consequences: Characteristics of outcome expectancies	Benefits of pharmacy profession services in terms of Quality of life gained, Lives saved, Values added, dollars saved, medication error, medication safety, medication burden, preventing emergency visits; preventing patients going into long-term care- PH, HCP		
		Goals: Implementation intention	Simple process and easily integration into their workflow- PH, CP Integration of medication review in different setting- HCP	Difference in parameter from different professional perspective- PH	
		Goals (autonomous)	Areas of relevant research studies (Access issues, affordability issues, side effects, important to a person around medication)- CP		**Incentivization** 1. Offer gift cards, free health screenings, or discounted pharmacy services for participation in medication reviews or studies 2. Provide financial compensation to pharmacists for their time and expertise in conducting medication reviews 3. Create performance-based incentives, such as bonuses for achieving measurable improvements (e.g., reduced medication errors, improved adherence 4. Provide medication management technologies (e.g., medication organizers, reminders) to enhance medication adherence 5. Award Continuing Professional Development (CPD) credits for pharmacists attending training or workshops 6. Offer financial incentives or recognition for clinics successfully implementing medication reviews 7. Provide financial stipends or honoraria for participating in extended research activities **Coercion** 1. Set policy mandates requiring medication reviews in specific settings (e.g., memory clinics) 2. Introducing institutional policies that require participation in medication reviews as part of professional duties (e.g., annual performance metrics 3. Implement consequences for non-compliance, such as decreased funding or limited access to professional development opportunities **Environmental restructuring** 1. Redesign workflows to include medication reviews as a routine process 2. Provide dedicated resources (e.g., space, tools, technology) for efficient integration 3. Implement standard operating procedures (SOPs) for medication reviews across various settings (memory clinics, FHTs, community pharmacies 4. Promote a team-based care model, where all professionals understand their roles in supporting medication reviews)
	Automatic motivation	Reinforcement	Adequate remuneration- PH, HCP		
		Emotion	Workload burden- PH, HCP	Participating in too many studies can be a barrier for this study- CP	
Opportunity	Physical opportunity	Environmental context and resources: Resources/material resources	Proper information and tools- PH Easy data collection tool and data coalition- PH Study coordinator (Tracking, organizing, managing the study)- HCP Interpretation services for patient or care partner whose first language is not English- CP Handout or material explaining about the study benefits- CP	Time consuming data collection- PH Complex data collection- PH Time commitment- HCP, CP Lack of resources- HCP Lack of access to pharmacist in the clinic- HCP	**Training** 1. Conduct training sessions on using data collection tools, ensuring ease of use and clarity for all participants 2. Provide education on study processes for healthcare professionals, including how to communicate effectively with non-English-speaking patients 3. Introduce pharmacist onboarding programs to familiarize them with clinic workflows and their role in the study **Environmental restructuring** 1. Develop and deploy easy-to-use data collection tools (e.g., mobile apps or automated systems) to simplify and expedite data entry
					2. Provide interpreters or multilingual resources (e.g., translated handouts) to assist patients and care partners whose first language is not English 3. Introduce telehealth platforms to conduct virtual medication reviews for patients unable to visit in person 4. Appoint study coordinators responsible for tracking, organizing, and managing study-related tasks to reduce participant and healthcare professional’s burden 5. Engage institutional stakeholders early to align approval timelines 6. Schedule regular interdisciplinary meetings to discuss roles, goals, and progress 7. Collaborate with provincial bodies to align study practices with local regulations and capacities 8. Establish regular interdisciplinary team meetings to improve communication among HCPs and resolve conflicts 9. Create comfortable and private spaces for patient-HCP interactions to build trust and encourage participation 10. Develop community engagement strategies to extend study benefits and awareness beyond the memory clinic **Enablement** 1. Supply participants and care teams with guides and checklists summarizing study processes 2. Provide templates for data collection and medication review reports to reduce complexity and ensure consistency 3. Enable participants to report challenges or suggest improvements, ensuring continuous process optimization **Restriction** 1. Ensure all data collection and medication reviews are conducted using pre-approved systems or forms 2. Encourage participants and professionals to focus on study-specific activities during designated periods 3. Address ageism explicitly by ensuring study materials and training emphasize the capability and contributions of older adults 4. Create protocols to limit intergroup conflict, such as requiring regular team updates and communication checkpoints **Persuasion** 1. Use impact stories that show how teamwork and adaptability lead to better patient outcomes and research success 2. Highlight the long-term benefits of the study for the clinic, such as improved workflows, funding opportunities, or enhanced care models 3. Conduct recognition events to reward and acknowledge team members who contribute to the study’s success **Incentivization** 1. Offer stipends or non-monetary incentives (e.g., recognition) to care partners for their time and participation
	Environmental context and resources: Organizational culture/climate	Flexibility of team as facilitator- HCP Research oriented team- HCP Research centered site- HCP Interdisciplinary work in memory clinic- PH, HCP, CP Good working environment for HCP and care partner- HCP, CP	Study approval process at institution- PH Staffing changes- HCP		
		Environmental context and resources: Facilitator	Integration of medication review in routine visit- CP		
		Environmental context and resources: Salient events		Working with the Family health team post-COVID- PH	
		Environmental context and resources: Environmental stressor		operational level barrier (Virtual vs in-person care; Not having same day appointment; Limited availability of pharmacist)- PH Care partner burden- CP	
		Environmental context and resources: Barrier		Increased workload due to medication review study- HCP Loss of support after the study- HCP Generalizability of study to other provinces- HCP Difference in pharmacist role from province to province- HCP Issues of capacity and consent in dementia vary from province to province- HCP	
	Social opportunity	Social influences: Social support	Connecting with right professionals- PH Messaging and communication about study- HCP Research team resolving ethics barrier- PH Comfort level of Patient with HCP- CP Support from family to participate in research- CP		
		Social influences: Social norm		Ageism- HCP Researcher perception about limited knowledge of patient related to research- CP	
		Social influences: Intergroup conflict		Lack of communication among doctors and with patients- CP	
			Study conducted in memory clinic will have ripple effect on the involvement of community- HCP		

COM-B Capabilities-Opportunities-Motivation-Behaviour; TDF Theoretical Domain Framework.

CP Care-partners and healthcare administrators.

PH Pharmacists and physicians.

HCP Social workers and occupational therapists.

### 3.1. Facilitators

#### 3.1.1. Domain 1: Knowledge.

Participants identified procedural knowledge of the study as a key facilitator for the implementation of the medication review study. Sufficient information about the study objectives, methods, expected outcomes and potential benefits were identified as a key facilitator.

“... How do you recruit patients? How do we record all that information as well? Like would there be a little nice flow chart?...” — RPH-006

“... if team members would be expected to report or document things but if they had a good understanding about... validity and reliability. So that when they were documenting whatever or transposing it, … to make a study valid they need to need to do it with accuracy …” — HCP-002

“... But I think that, you know, if you ask and say, OK, you know, this is, you know what we are doing, this is how we do it. Do you want some more information. This is where you can find information. Can I answer any of your questions. So if it’s being an inclusive team, you don’t necessarily need to give a lot of information”— CP-003

Designing a study with a simple process that allows for easy integration of study procedures into pharmacists’ workflow can significantly enhance the ease and effectiveness of the research.

“I just like to add that, I mean, likely if it is all of us doing it …, just to have it really efficient. We’re busy...is not the only thing we’re doing all day. So, something really simple that would be part of our daily process would be ideal and that could facilitate the work”— RPH-001

Moreover, HCP and CP stakeholders believed that making the study design and procedures simple would act as a key facilitator for the patient. The CP stakeholders indicated that participating in the study should be easy for them, and they should not require any new skills to participate.

“So … if you’re recruiting people living with dementia and family, just to be aware of that burden and to make it as easy as possible to participate and to … make it really clear what the value is … to motivate them to participate.”— HCP-005

“I would hope that this study would be brought to the level of people participating that would need no more skills....”— CP-001

#### 3.1.2. Skills.

PH and HCP groups noted that specialized training for conducting medication reviews would facilitate the implementation of the study. They emphasized that, to accurately assess the impact of medication reviews as an intervention, it is essential that pharmacists are proficient in conducting these reviews with PWCID, and that they possess the necessary skills and knowledge to do so. Participants highlighted that effective training and education in medication review processes will enable pharmacists to implement this intervention correctly.

“… you have to train pharmacist… there’s the technical know-how. So, it’s standardized and it’s an understanding that expertise is used. If you have pharmacists who are interested and have not undergone the training, to define the tools they need to qualify through or have to include them in the study as … pharmacists for this [Clinic Name] work.”— RPH-002

#### 3.1.3. Decision processes.

One of the key facilitators identified by the CP group was their desire to be actively involved in the study and decision-making process, rather than just being a participant in a research study. Many care partners perceived that when their voices are heard and integral to a study’s decision-making process, it fosters a sense of empowerment, engagement, and trust. Moreover, care partners believed that including patients and care partner as active members of the research team, rather than just as participants, can significantly enhance the relevance, quality, and impact of the research.

“… the culture needs to change to where patients or care partners are part of the research team. And perhaps when … care partners or patients feel that their voices are going to be heard or they are part of the decision making, part of the study. And … looking at it from the patient-care partner lens, I think there will be more … response. … I would want to be part of this study because I would feel that like it would be looking at it from a patient or care partner lens.”— CP-003

“I think there are benefits, … rather than having patients and care partners as participants, … try and include them to be part of the research team so that you even that the questions that are being asked are more relevant. And even when they’re interpreting the results, they are like they’re looking at it from a patient point of view.”— HCA-002

#### 3.1.4. Social/professional role and identity.

The pharmacists’ knowledge and skills could contribute to the efficiency and smooth execution of the study, making it easier for all involved. HCPs believed an interdisciplinary team could significantly facilitate and enhance the work involved in various projects, including research and patient care. This positive perception underscores the importance of collaborative, interdisciplinary approaches in designing and conducting studies that aim to improve patient outcomes, particularly in the realm of medication management.

“Ontario health team is ... really motivated and pushing interdisciplinary work, which memory clinics are and which this kind of study ... would encourage and foster. So that could be in your favor as well.”— HCP-005

#### 3.1.5. Beliefs about capabilities.

Participants highlighted that pharmacists are “essential” and “important members” of the memory clinic model. They add significant value, contributing to comprehensive patient care and improved outcomes for individuals with memory-related disorders, such as Alzheimer’s disease and other forms of dementia. Pharmacists bring specialized expertise that enhances the multidisciplinary approach in memory clinics.

Participants noted that getting recognition and acknowledgment of pharmacist work in memory clinics would be a facilitator for the study in getting more support and resources for the study. It is crucial to highlight their contributions and reinforce their value to patient care.

“And this could be a really good opportunity to show, actually, if you give pharmacists the proper tools and information, they can actually make meaningful contributions...”— RPH-003

“The physicians really look to pharmacists to help make decisions about what to prescribe. The pharmacists are good at taking that physical exam, all their medical assessments, and guiding the physician sometimes. It’s an amazing collaboration... They piece it all together in the team approach, and the outcome happens” — HCP-002

#### 3.1.6. Optimism.

CP focus group showed a positive attitude towards a large-scale medication review study, and they believed it would be of benefit to showcase improvements that can be made to increase effectiveness for PWCID and care partner. Care partners who believe in the benefits of the study are likely to share their positive experiences and recommendations with other potential participants. This word-of-mouth promotion can significantly contribute to a higher level of interest and engagement among potential recruits. A positive attitude can increase the overall willingness of care partners to engage with the research process. This may include being more open to providing informed consent, attending study-related sessions, and actively participating in data collection, thereby streamlining the recruitment process.

“We’re going to see people who are very interested in advancing research... knowing that it will benefit someone in the future.”— CP-001

#### 3.1.7. Beliefs about consequences.

All three focus groups showed belief in the effectiveness of medication reviews and its impact on inappropriate medication, and was identified as a key facilitator in this domain. The shared belief in the effectiveness of medication review across diverse stakeholder groups signifies a unified perspective on the potential positive impact of this intervention.

“I think of the condition we’re kind of trying to help with MCI [mild cognitive impairment]...when we’re looking at things that’ll interfere with the patient’s quality of life, I think medications could be traumatic day to day...coming off of it and seeing benefits. So, I think medications might have the biggest possible impact on the patient quality of life in this population..”— RPH-005

“I think ... it’s important because the trajectory of dementia changes and not everybody requires medication at all times, but medication impacts upon the experience of dementia, both from a care partner’s point of view as well as the person’s point of view.”— HCA-002

In addition, PH stakeholders mentioned other characteristics of outcome expectancies, such as quantifying their services in terms of money saved, reduced medication errors, reduced medication burden, and increased medication safety and affordability. HCPs believed that showing the benefits of medication review will be a facilitator in the future for funding other research and pharmacist integration within healthcare teams. Examples of impactful outcomes to be measured that were identified by this group included preventing hospitalization and emergency visits, lessening the number of patients from going into long-term care and reducing adverse events.

“...we have the medication piece where you say unnecessary medication or medication burden because of anticholinergic load, and we can quantify a number of pills per day saved, dispensation cost saved. So, cost minimisation analysis add that piece and then we can go for other analysis and say, what does this mean in quality of life … reduction of care partner burden or taking time away”— RPH-002

“That seems to be the aim is to keep people out of emergency department and keep an eye out of long-term care longer. So can you demonstrate... and use it for further funding... to demonstrate that they’re meeting the goals of ministry.”— HCP-004

#### 3.1.8. Goals.

Participants noted that it is crucial to ensure that patient-identified goals and study outcomes are integrated into the healthcare plan and research study. Integrating these goals is fundamental in creating a patient-centered approach to treatment, where the focus is not only on medical outcomes but also on aligning care with the individual’s values, preferences, and desired quality of life. These parameters provide a common ground for collaboration and decision-making between healthcare providers and patients. Not setting autonomous goals from the patient’s perspective can be a barrier to the full-scale study while setting patient-identified goals can serve as a facilitator for a study in various ways, enhancing motivation, engagement, and the overall success of the research.

“Parameters that are again … important to all the clinicians, but also to the patient, because I think you know we can come … with parameters that either make us look good or make us look bad, but … has no patient impact …”— RPH-005

“So are there access issues? Are there affordability issues? Are there side effects? … you know what’s important to a person around medication?”— HCA-002

#### 3.1.9. Environmental context and resources.

Interdisciplinary work at MINT memory clinics, research-oriented teams, and research-centered sites were mentioned as a key facilitator of such a study.

“it also comes down some centers have a very research focus like that’s their mandate. That’s what they ‘re looking for projects because they have to show some research involvement. So, I think some are research centered, some just recognize the value of it.”— HCP-002

### 3.2. Barriers

#### 3.2.1. Social/professional role and identity.

Although non-pharmacist members contribute to medication management among older adults, clear and precise roles for interdisciplinary staff in pharmacist-led medication review studies are crucial for ensuring the intervention’s consistency and effectiveness across different sites.

“Another barrier could be … other interdisciplinary staff other than pharmacists, don’t know whether they belong in a pharmacy study … But I think, um, sometimes as a social worker, I feel that I don’t belong in the pharmacy world, although I have things to contribute as far as medications and medication compliance.” — HCP-005

#### 3.2.2. Reinforcement.

The lack of remuneration can be a potential barrier for clinician team members when invited to participate in studies. Pharmacists and other healthcare professionals believed they should be adequately remunerated for their role in the study.

“I just was saying... pay people like pay well, build that in, adequate. We pay patients and care partners, which is necessary. You’ll need to apply for enough money to make it financially worth. If you want them to actually participate because it’s just too busy in a clinic as it is... so build-in adequate funding for everybody.”— MPP-001 (Moderator)

#### 3.2.3. Emotion.

PH highlighted that they are already working at full capacity and overwhelmed with clinical responsibilities so adding research tasks can decrease their interest in participating. CP group also mentioned that participation in too many studies can cause participation fatigue and burden their clinical visits.

“I mean, for sure, like I would want to not even want to go to the memory clinic if every now and then someone else is coming to me for a study, I’m going to get to a point where I’ll say, you know what? Forget this memory clinic.”— CP-003

“I support [Lead Name] in saying my director saying it’s all about capacity and we are all at the end of the rope. So those are the keywords.”— RPH-002

#### 3.2.4. Environmental context and resources.

Pharmacists highlighted that time-consuming and complex data collection, difficult study approval processes at their institution, and operational level barriers such as limited availability of the pharmacists could present significant barriers for pharmacists to be involved in research.

“Meds review um and that [web application] was the biggest barrier for me. …, it was just like you had to log on. You had to put in all this information you have to figure out. And so, you’re seeing the patient and then... inputting all that data afterwards.”— RPH-006

Participants also noted that time commitment, resources needed for the study, lack of access to pharmacist in the clinics, increased workload due to full-scale medication review study, loss of support or resources after the study, and lack of generalizable study procedures to other provinces of Canada could be significant potential barriers.

To reduce these barriers, participants recommended key enablers, such as employing a study coordinator at each research site to be responsible for overseeing study procedures. Addressing the potential barriers identified by healthcare professionals involve considering both scenarios—existing MINT pharmacists and pharmacists added specifically for the study—separately, as each present distinct challenges and solutions. For clinics where pharmacists are already integrated into the clinics, the challenges primarily revolve around time commitment, resource allocation, and increased workload due to the demands of a full-scale medication review study. These pharmacists are already integrated into the clinical workflow, which means their involvement in research could strain existing resources further. On the other hand, introducing new pharmacists solely for purpose of conducting a study introduces challenges related to integration into the clinical team and sustainability post-study.

“I think the disadvantages you have; you find out what a pharmacist can add and then study ends and you’re without it. So, I’ve done a few different clinics. There was one had a just different or lesser pharmacy role, and it was tough to go from what I was used to something to something less comprehensive.”— HCP-002

Time commitment from PWCID and their and care partners and caregiver burden were also noted as a barrier.

“I think of the families I support, the care partners, I know the first thing they would say is I cannot take on any more responsibility. I’ve got enough as it is for me as an outside care partner... But there all those care partners, a whole bunch just exhausted. So, I could understand that would be a real barrier.”— CP-001

Furthermore, availability of services to ensure participation by diverse populations was also highlighted as a barrier. For example, the availability of translation services for non-English speaking individuals is lacking in many healthcare institutions. Addressing language barriers through such services is essential to ensure equitable access and effective communication, enabling inclusive and culturally competent healthcare research.

“I the only thing I was thinking about was ... interpretation services. So, if somebody’s … first language isn’t the language that’s being used at the memory clinic, then it would be important to account for that.”— HCA-002

#### 3.2.5. Social Influences.

Some care partners discussed the influence that their own families had on their participation. A care partner described family support as crucial in encouraging and facilitating an individual’s participation in research studies.

“The first thing he said was go, I mentioned it to my second son and the third, and they said, definitely go mom. We want you to. So...our family there was no hesitation or question.”— CP-002

### 3.3. Implementation recommendations for behavior intervention techniques

The facilitators and barriers identified in the analysis of interviews were mapped to behavior change intervention techniques on the BCW to facilitate the implementation of research (see Table 2). Capability barriers, such as limited knowledge of study processes, among healthcare professionals can be mitigated through standardized training programs, tailored educational workshops and case and scenario-based training that focuses on both general and patient-specific challenges. Incorporating hands-on training, role-play exercises, and user-friendly tools like templates, visual aids and flowcharts in multiple languages can simplify complex study procedures. Motivational barriers may be mitigated by highlighting pharmacists’ roles, providing continuing professional development (CPD) credits or incentives, and showcasing successful initiatives. Opportunity barriers related to resource limitations can be managed by deploying efficient tools like digital data collection platforms, establishing telehealth services, and providing multilingual resources to accommodate diverse patient populations. Social opportunity barriers, such as communication gaps, can be reduced by fostering collaboration, leveraging team-based care models, and using structured group activities.

## 4. Discussion

In this qualitative study, we used the TDF to identify potential barriers and facilitators that have the potential to impact the implementation of research projects designed to examine the impact of medication reviews in PWCID. The use of TDF enabled systematic determination of important factors that may influence the implementation of the research in MINT memory clinics [26]. The tailored interview guides and inclusion of clinicians, care-partners, and healthcare administrators ensured that the study explored a comprehensive and holistic range of facilitators and barriers [27].

Facilitators identified included the importance of procedural knowledge about the study, ensuring stakeholders are well-informed about the study’s objectives, methods, and expected outcomes, specialized training for pharmacists and healthcare professionals conducting medication reviews, involving care partners and patients as active decision-makers in the study, and recognizing the valuable role of pharmacists in memory clinics, which enhances comprehensive patient care. On the other hand, several barriers were also identified, such as the lack of will to conduct the medication review study, potential burnout among healthcare professionals [28], PWCID and care-partners, and challenges like complex data collection, institutional approval processes, and limited availability of pharmacists. Additionally, resource-related barriers, including the need for adequate remuneration and time commitments, were highlighted, as well as the need for a simple study design and the importance of interdisciplinary collaboration in memory clinics.

In a systematic review conducted by Paksaite et al to identify barriers and facilitators to the adoption of prescribing guidelines, one of the dominant barriers was lack of time [29]. While financial incentives can help address the barrier of lack of time in adopting prescribing guidelines, they are not the sole solution. Streamlining workflows through user-friendly tools, such as decision support systems integrated into electronic health records, can save time by simplifying guideline implementation. Providing targeted education and training, including concise summaries or decision aids, ensures that healthcare providers can quickly understand and apply guidelines. Additionally, fostering interdisciplinary collaboration, where tasks are shared among team members such as pharmacists and nurses, can effectively distribute the workload [30]. The identified lack of remuneration as a barrier in our study aligns with the broader discourse on financial incentives within the healthcare landscape. The findings underscore the significance of adequately compensating healthcare professionals and stakeholders involved in research. The absence of remuneration is perceived as a potential hindrance, particularly among professionals already grappling with a demanding workload and potential burnout [31,32]. We compared our findings with the systematic review by Paksaite et al. because, despite focusing on the adoption of prescribing guidelines, it examines similar interprofessional dynamics, workflow challenges, and contextual factors relevant to implementing medication-related interventions. Both settings involve pharmacists, time constraints, communication processes, and interdisciplinary roles, making the review a relevant comparator for understanding broader system-level barriers and facilitators.

Lack of awareness and understanding of study procedures was also a barrier identified in both our findings and the systematic review conducted by Paksaite et al [29]. Recognizing the importance of knowledge about the study, it’s evident that disseminating information alone may not be sufficient, but patients should be directly and actively involved in the study design and identification of important outcomes. Demonstrating the effectiveness and potential beneficial outcomes of pharmacist-led interventions could significantly enhance care practices for PWCID. This realization not only solidifies the importance of the research but also serves as a compelling factor to garner interest and encourage participation from various stakeholders, including healthcare professionals, researchers, patients, and their families. Stakeholders believed that positive results from the study may help in getting more funding for research. Quantifying the pharmacist’s professional services in terms of cost-effectiveness, reduced medication error, reduced medication burden, increased medication safety, and affordability can assist in getting more support, resources, and funding for the study and memory clinics. The high frequency of medication error and inappropriate prescribing is a significant problem for healthcare systems and frequently contributes to adverse drug events, many of which are avoidable [33].

The study delves into the social influences and support networks surrounding persons with dementia. Care partners highlight the crucial role of family support in encouraging individuals to participate in research studies. This finding underscores the interconnectedness of social networks in influencing healthcare decisions for persons with dementia [34]. Understanding and leveraging these social dynamics can contribute to more effective medication reviews, ensuring that interventions align with the broader context of the patient’s social support system. The study also touches upon the beliefs and expectations of stakeholders regarding the capabilities and consequences of medication reviews. Pharmacists and healthcare professionals express confidence in the positive effects of these interventions, emphasizing the potential impact on patients’ quality of life. Care partners exhibit a positive attitude towards the study, anticipating benefits for both care partners and patients. Exploring these beliefs provides insights into the collective optimism surrounding medication reviews, serving as a driving force for engagement and collaboration among stakeholders.

The evaluation of medication reviews in individuals with cognitive impairment and dementia is shaped by a complex interplay of factors involving diverse stakeholders, varied perspectives, and broader healthcare considerations. Our TDF analysis highlights the importance of a study coordinator. We can tailor future interventions to address barriers and gaps in current multidisciplinary teams. If a potential barrier is related to social influences, then strategies to increase motivation and engage influential individuals can be integrated into the intervention design. If social support and peer influence are facilitators in a specific domain, then we can incorporate these factors into the intervention by incorporating group sessions or peer mentoring [35].

Recruiting participants for research involving PWCID is inherently challenging, as highlighted in prior studies [36,37]. Factors such as caregiver burden, patient reluctance, and logistical barriers often impede recruitment efforts [38–40]. For example, Davis et al. noted that recruitment rates were significantly lower in populations requiring complex care compared to general clinical populations. The review highlights key factors impacting the recruitment of older adults with dementia for research. Barriers include gatekeepers, such as caregivers and healthcare providers, who may restrict access due to time constraints, risk concerns, or lack of interest [36]. Mistrust in research processes and non-targeted recruitment methods, like general advertisements, further hinders participation. Facilitators for successful recruitment emphasize the importance of community partnerships and trust-building, particularly in engaging minority populations. Additionally, employing multiple recruitment strategies, such as direct referrals and targeted outreach, and offering incentives like free cognitive screenings, can significantly enhance recruitment efforts and ensure more representative participation in research [36].

Our study mapped key challenges, such as limited knowledge of study procedures (Capability), time constraints and resource limitations (Opportunity), and concerns about workload burden and professional recognition (Motivation), to targeted interventions. Strategies included education and training to enhance knowledge, workflow restructuring and digital tools to reduce operational burdens, and persuasion, incentivization, and modeling to improve pharmacist engagement. These findings align with McCarthy et al. , who applied the BCW framework to develop a targeted intervention. Using the COM-B model, the study identified critical behaviors for healthcare providers—“Ask, Investigate, and Deprescribe” (A-I-D)—to address prescribing cascades [41]. Barriers, such as lack of knowledge (Capability), organizational constraints (Opportunity), and fear of patient harm (Motivation), were systematically assessed. These insights were mapped to intervention functions, including education, enablement, and persuasion, and paired with behavior change techniques (BCTs) to create actionable strategies. The study highlights the BCW’s utility in tailoring interventions to promote safe prescribing practices, reduce unnecessary medications, and improve patient outcomes in primary care settings [41].

To the best of our knowledge, this was the first study that has explored the potential barriers and facilitators using the TDF to evaluate the impact of medication reviews in PWCID in primary care-based memory clinics. The major strength of this study was the diverse sample, including patients and care partners, clinicians, pharmacist, community workers. Another highlight of the study was the use of TDF to construct the interview guide and data analysis. When designing an intervention using the TDF, the roles of potential barriers and facilitators are integral to ensuring the intervention’s effectiveness. The TDF helps identify potential barriers by analyzing the different domains that could hinder behavior change and intervention implementation. The findings are specific to memory clinic settings and may not be directly transferable to other clinical or organizational contexts. Unlike one-on-one interviews, focus group discussions may not always allow all participants to express their thoughts and experiences in-depth. Some participants may be more reserved or overshadowed by dominant voices in the group. Additionally, we were not able to recruit any PWCID and gain their perspectives on medication review effectiveness. Care partners may have their own biases and perspectives that may not fully align with those of the individuals they care for. This study was limited by its single-day design, which did not allow for the recruitment of sample large enough to determine data saturation; some barriers and facilitators may have been missed. The use of virtual sessions and topic presentations prior to interviews to ensure a shared understanding of medication reviews may have unintentionally introduced social desirability bias. Despite our efforts to create an open and inclusive environment, mixing participants from different professional roles (e.g., physicians with pharmacists, and caregivers with administrators) may still have introduced hierarchical or relational bias, potentially limiting how openly some individuals shared their experiences.

## Conclusion

This study highlights the complex interplay of factors that influence the assessment of medication reviews in individuals with cognitive impairment and dementia. The findings highlight the importance of tailored interventions that address specific barriers and leverage facilitators to ensure the implementation of research of medication reviews in memory clinics is effective. This study adds to the limited evidence on implementing research related to pharmacist-led medication reviews in primary-care memory clinics by identifying specific behavioural and system-level barriers and facilitators using the TDF–COM-B approach. These insights can guide policymakers and healthcare administrators in designing targeted supports—such as clearer role definitions, resource allocation, and standardized training—to enhance the feasibility, sustainability, and scale-up of medication review interventions for PWCID. Future research should identify intervention components for targeting behavior within the selected domains and seek to select those components that are more likely to be relevant and feasible.

## Supporting information

S1 Table Barrier and facilitator questions for Clinicians (Pharmacists, Physicians, healthcare professionals (HCP). (PDF)

S2 Table Barrier and facilitator questions for Patients/Care Partners. (PDF)

S1 Appendix COREQ (COnsolidated criteria for REporting Qualitative research) Checklist. (PDF)
