# Supplementary material for: Barriers and facilitators to implementation research on pharmacist-led medication reviews in memory clinics: A qualitative study using the TDF-COM-B
Source: PLoS One. 2026 Jan 20;21(1):e0341014. doi: 10.1371/journal.pone.0341014 (PMC12818622; doi:10.1371/journal.pone.0341014)
Supplement: S1 Table — (PDF) [file pone.0341014.s001.pdf]

S1 Table: Barrier and facilitator questions for Clinicians (Pharmacists, Physicians, healthcare professionals (HCP)

Domain	Potential Questions
Motivation/Goals/Behavioural Regulation	Why do you think we should or should not do a study examining medication reviews and use in memory clinics? Do you think examining medication use and reviews should be a routine part of memory clinics?
Beliefs about capabilities	Do you think doing a study about medication use and reviews in memory clinics will be easy or difficult to do? Explore (if difficult what makes it difficult, if not difficult why?)
Skills	What skills/training do you think will be required to implement a study about medication use and reviews in memory clinics?
Beliefs About Consequences	What are the benefits or advantages of conducting such a study about medication reviews and medication use? What do you think are the disadvantages of doing such a study?
Memory/Attention/Decision	What would impact doing study procedures becoming routine in your institutions? To what extent do resources (e.g. time available, staff availability, patient/carer cooperation, etc) influence whether this study could be implemented successfully? How does that affect how willingness to implement such a study in your memory clinic?
Social Influences	In what way do patients/carer/family influence your decision about implementing such a study in your institution? Whose opinions would need to be considered before implementing such a study in memory clinics? Who would need to be involved in decision-making about the study?
Emotion	Is conducting a study such as this a difficult situation to deal with? Is it something you would prefer to avoid?
Professional Role	Do you think it is an appropriate part of your role to undertake a study about medication use and reviews or do you think another healthcare provider should be responsible for this? What kind of collaboration between different HCPs is necessary?
Integration and Implementation	What additional resources, either material or personnel, are required to implement such a study in a memory clinic? How about operational guidelines or responsibility of different parties? Do you feel such a study will be too complex to be implemented in your memory clinic or memory clinics in general? Whose responsibility would it be to implement such a study in a memory clinic? (Explore financial or human resources, clarity on operational guidelines, responsibilities for implementation, conflicts with other policies, coordination, collaboration between parties, motivation, political will)
