# Supplementary material for: Barriers and facilitators to implementation research on pharmacist-led medication reviews in memory clinics: A qualitative study using the TDF-COM-B
Source: PLoS One. 2026 Jan 20;21(1):e0341014. doi: 10.1371/journal.pone.0341014 (PMC12818622; doi:10.1371/journal.pone.0341014)
Supplement: S2 Table — (PDF) [file pone.0341014.s002.pdf]

S2 Table: Barrier and facilitator questions for Patients/Care Partners

**Domain**	**Potential Questions**
Motivation/Goals/Behavioural Regulation	Do you think we should or should not do a study examining medication reviews and use in memory clinics? Do you think examining medication use and reviews should be a routine part of memory clinics?
Beliefs about capabilities	Do you think doing a study about medication use and reviews in memory clinics will be easy or difficult to do? Explore (if difficult what makes it difficult, if not difficult why?)
Skills	What skills/training do you think you or the person with dementia you care for will require to participate in a study about medication use and reviews in memory clinics?
Beliefs About Consequences	What are the benefits/advantages of conducting such a study about medication reviews and medication use? What do you think are the disadvantages of doing such a study?
Memory/Attention/Decision	To what extent do resources (e.g. time available, patient co-operation, etc) influence whether you would participate in such a study?
Social Influences	In what way does the patient or other family members influence your decision about participating in such a study? Whose opinions would need to be considered before you participate in such a study?
Emotion	Is participating in a study such as this be a difficult situation to deal with? Is it something you would prefer to avoid?
